# Silver Nanoparticles Functionalized With Antimicrobial Polypeptides: Benefits and Possible Pitfalls of a Novel Anti-infective Tool

**DOI:** 10.3389/fmicb.2021.750556

**Published:** 2021-12-17

**Authors:** Maria S. Zharkova, Olga Yu. Golubeva, Dmitriy S. Orlov, Elizaveta V. Vladimirova, Alexander V. Dmitriev, Alessandro Tossi, Olga V. Shamova

**Affiliations:** ^1^World-Class Research Center “Center for Personalized Medicine”, FSBSI Institute of Experimental Medicine, Saint Petersburg, Russia; ^2^Laboratory of the Nanostructures Research, Institute of Silicate Chemistry, Russian Academy of Sciences, Saint Petersburg, Russia; ^3^Department of Life Sciences, University of Trieste, Trieste, Italy

**Keywords:** nanoparticles, antimicrobial peptides and proteins, antibacterial activity, drug-resistant bacteria, cytotoxicity

## Abstract

Silver nanoparticles (AgNPs) and antimicrobial peptides or proteins (AMPs/APs) are both considered as promising platforms for the development of novel therapeutic agents effective against the growing number of drug-resistant pathogens. The observed synergy of their antibacterial activity suggested the prospect of introducing antimicrobial peptides or small antimicrobial proteins into the gelatinized coating of AgNPs. Conjugates with protegrin-1, indolicidin, protamine, histones, and lysozyme were comparatively tested for their antibacterial properties and compared with unconjugated nanoparticles and antimicrobial polypeptides alone. Their toxic effects were similarly tested against both normal eukaryotic cells (human erythrocytes, peripheral blood mononuclear cells, neutrophils, and dermal fibroblasts) and tumor cells (human erythromyeloid leukemia K562 and human histiocytic lymphoma U937 cell lines). The AMPs/APs retained their ability to enhance the antibacterial activity of AgNPs against both Gram-positive and Gram-negative bacteria, including drug-resistant strains, when conjugated to the AgNP surface. The small, membranolytic protegrin-1 was the most efficient, suggesting that a short, rigid structure is not a limiting factor despite the constraints imposed by binding to the nanoparticle. Some of the conjugated AMPs/APs clearly affected the ability of nanoparticle to permeabilize the outer membrane of *Escherichia coli*, but none of the conjugated AgNPs acquired the capacity to permeabilize its cytoplasmic membrane, regardless of the membranolytic potency of the bound polypeptide. Low hemolytic activity was also found for all AgNP-AMP/AP conjugates, regardless of the hemolytic activity of the free polypeptides, making conjugation a promising strategy not only to enhance their antimicrobial potential but also to effectively reduce the toxicity of membranolytic AMPs. The observation that metabolic processes and O_2_ consumption in bacteria were efficiently inhibited by all forms of AgNPs is the most likely explanation for their rapid and bactericidal action. AMP-dependent properties in the activity pattern of various conjugates toward eukaryotic cells suggest that immunomodulatory, wound-healing, and other effects of the polypeptides are at least partially transferred to the nanoparticles, so that functionalization of AgNPs may have effects beyond just modulation of direct antibacterial activity. In addition, some conjugated nanoparticles are selectively toxic to tumor cells. However, caution is required as not all modulatory effects are necessarily beneficial to normal host cells.

## Introduction

Antibiotics have become a cornerstone of modern medicine and health care. Their use made it possible not only to successfully treat bacterial infections but also to safely perform a variety of medical procedures that carry a high risk of infection by opening pathways for microbes to enter the body and/or weaken our immune defenses. The ever-worsening antibiotic resistance crisis is a major medical, scientific, and governmental concern worldwide (Barriere, 2015; Ventola, 2015; World Health Organization, 2015; Cassini et al., 2019). The problem is exacerbated by these molecules’ own success as highly efficient antibacterial agents, which has led to large scale and inappropriate use in non-medical settings, for example, as prophylactic agents for industrial livestock in densely stocked closed environments or, to a lesser extent, even for food preservation. This has contributed significantly to the spread of resistance.

The need for new antimicrobial agents is obvious. The ideal scenario would be to find new effective compounds that are both impervious to the current bacterial resistance profile and that can break the resistance cycle, or at least delay the time of its development after the new drug is widely used. A low propensity to induce bacterial resistance is favored by a variegated antimicrobial mechanism, preferably not requiring the presence of specific targets, and a steep time-kill curve (Zimmerman et al., 2007; Wimley and Hristova, 2011; Hemeg, 2017; Yu et al., 2018; León-Buitimea et al., 2020). Antimicrobial peptides (AMPs) or their derivatives represent a promising group of compounds that meet these requirements. They are essential molecular tools of eukaryotic host defense, found throughout the eukaryotic kingdom from protists to multicellular animals and plants (Li et al., 2012; Pasupuleti et al., 2012). In higher vertebrates, they act as innate immune effectors, directly inactivating microbes within the granules of phagocytic cells, or when secreted by granulocytes, epithelia and various glands (Tosi, 2005; Lai and Gallo, 2009). Once in the intercellular space, AMPs may also exert immunomodulatory effects, participate in opsonization and chemoattraction processes, and promote wound healing and angiogenesis by stimulating cellular proliferation (Lai and Gallo, 2009; Riera Romo et al., 2016; Mookherjee et al., 2020).

AMPs are generally cationic and amphipathic molecules; this increases their affinity for the bacterial surface, which tends to be anionic, and facilitates their insertion into the bacterial membrane (Matsuzaki, 2009; Takahashi et al., 2010; Rahnamaeian, 2011; Teixeira et al., 2012). Indeed, bacterial membranes are the initial and often the primary target for most AMPs, which compromise their integrity by forming small lesions or nonselective pores, or even by completely disrupting the phospholipid bilayer in a detergent-like manner, resulting in the leakage of ions and other essential metabolites. They can also initiate lipid segregation and other types of microenvironmental perturbations around essential membrane-associated protein machinery and impair its function. Some AMPs can translocate across the membrane into the bacterial cytoplasm by a self-promoted uptake mechanism, or less commonly by using specific transporters, and then act intracellularly by targeting essential processes such as transcription and protein synthesis (Brogden, 2005; Nguyen et al., 2011; Graf and Wilson, 2019; Huan et al., 2020).

Another promising group of candidates as antimicrobial therapeutics are various types of nanomaterials, in particular silver nanoparticles (AgNPs; Makowski et al., 2019; Fernandez et al., 2021; Spirescu et al., 2021). The antimicrobial properties of silver were known to mankind since long before the germ theory of disease was formulated or bacteria observed (Alexander, 2009). The exact mechanism behind the antimicrobial action of silver in nanoparticles, and whether it acts in a free ionic or nanoparticulate form, has not yet been elucidated in all its complexity. The known aspects clearly indicate a multifaceted capacity to rapidly and effectively disrupt a number of vital functions in the bacterial cell (Rai et al., 2012; Mikhailova, 2020). The antimicrobial activity of AgNPs depends on their shape, form and size, and despite the fact that many of the observed effects are attributed to the release of Ag^+^ ions from their surface (Tang and Zheng, 2018), the modes of action of AgNPs and Ag^+^ ions are not completely overlapping (Durán et al., 2010; Kędziora et al., 2018).

Silver nanoparticles, like Ag^+^ ions, have a high affinity for anionic compounds (Kim et al., 2007; Dakal et al., 2016; Mikhailova, 2020), so their attraction to bacterial cells has the same electrostatic basis as that of AMPs. Once adhered to the bacterial surface, AgNPs induce a “pitting” effect in the peptidoglycan layer and cause membrane disordering, leading to leakage and dissipation of the transmembrane potential as well as damage to membrane-associated proteins (Dakal et al., 2016; Tang and Zheng, 2018; McNeilly et al., 2021). One of the main foci of disruption is the respiratory process in bacteria, as AgNPs can seriously impair the functioning of respiratory chain. This not only cuts off the cell’s energy supply but also induces the production of toxic reactive oxygen species (ROS) that contribute to cellular damage (Holt and Bard, 2005; Dakal et al., 2016). Moreover, the resulting increase in molecular O_2_ at the bacterial surface stimulates oxidative dissolution of AgNPs, promoting the release of toxic Ag^+^ ions (McNeilly et al., 2021).

While larger AgNPs tend to remain on the bacterial surface, smaller ones, as well as the released ionic Ag^+^, have been shown to penetrate cells. Here, they additionally affect (i) nucleic acids (AgNPs can cause DNA fragmentation, while Ag^+^ initiates DNA condensation); (ii) various essential enzymes and structural proteins (Ag^+^ forms complexes with thyol, amine or other electron donor groups, essential for protein function); (iii) cell signaling cascades (which rely heavily on phosphorylation/dephosphorylation “switching” and are therefore affected by the electrostatic attraction between AgNP/Ag^+^ and phosphate groups; Dakal et al., 2016; McNeilly et al., 2021).

Both AMPs and AgNPs have demonstrated their potential in combating resistant microbes (Lara et al., 2010; Rai et al., 2012; Roque-Borda et al., 2021), and both have also been found to act synergistically with conventional antibiotics (Cassone and Otvos, 2010; Wan et al., 2016; Krychowiak et al., 2018; Ruden et al., 2019; Zharkova et al., 2019; Duong et al., 2021). Moreover, it has previously been reported by us (Zharkova et al., 2019) as well as by others (Ruden et al., 2009; Salouti et al., 2016), that AMPs and AgNPs can enhance each other’s antimicrobial activity, when used in combination. In this paper, we want to go a step further and apply these compounds not just in association, but as a conjugated complex. It is known that surface coating can increase the colloidal stability and bioavailability of AgNPs while decreasing their immunogenicity. Using different types of biomolecules, including peptides or proteins, for such a task is a common approach to enrich or refine the biological properties of NPs (Heuer-Jungemann et al., 2019; Makowski et al., 2019). From the perspective of antimicrobial peptides (AMPs) or antimicrobial proteins (APs), binding to the surface of NPs is also one of the suggested ways to improve stability under physiological conditions (Casciaro et al., 2020; Dijksteel et al., 2021). Here, we investigate whether AgNP-AMP/AP conjugates are successful in terms of antimicrobial capacity and selectivity toward eukaryotic cells, and how their binding alters the activity of each component.

## Materials and Methods

### Preparation of AgNPs and Their Conjugates With Antimicrobial Peptides and Proteins

The AgNPs and AgNP-AMP/AP conjugates investigated in this work were obtained using the photo-reduction method. Despite some drawbacks, this type of physical method for NP production limits the presence of unwanted residual components such as synthesis reagents or pyro- and immunogenic molecules (e.g., LPS), as is the case for some green synthesis scenarios, which may alter the results of activity testing (Iravani et al., 2014; Fernandez et al., 2021).

The AgNPs used as the core possessed an envelope consisting only of gelatin and were the same as used previously for synergistic effects with free AMPs and lysozyme (Zharkova et al., 2019). For the present study, we chose the following (poly)peptides with antimicrobial properties for preparing AgNP conjugates: porcine protegrin-1 (AMP: PG-1), bovine indolicidin (AMP: Ind), hen egg-white lysozyme (AP: Lyz), salmon protamine (AP: Prot), or calf thymus histones (AP: His). All conjugates were synthesized according to the protocol previously developed for the lysozyme/gelatin-coated AgNPs (Patent of the Russian Federation N° 2502259), with minor adjustments. Reaction mixture, containing 0.043–0.045% (m/m) of the AMP/AP, 0.073–0.085% (m/m) of AgNO_3_ (Chimmedsynthesis, Russia 99.9%), 0.071–0.080% (m/m) of gelatin (Acros, Russia), and H_2_O (the rest), was UV irradiated for 60 min at 254 nm and room temperature (RT) with constant stirring. The control AgNPs were synthesized by the same procedure using only gelatin without the AMP/AP component. AgNPs and conjugates were then dialyzed against H_2_O (1:500 v/v; H_2_O was changed once per 15 h) for 30 h at RT using 25 kDa molecular weight cut-off membrane to remove unbound proteins, peptides, and ionic silver. The low level of the latter was verified by the ionometry. The samples were shown to be stable in physiological NaCl solution. The thickness of the coating was judged to be about 12–15 nm, and average diameter of the whole particles ~50 nm, by transmission electron microscopy (see Supplementary Material).

### Antibacterial Activity

#### Bacterial Cells

*Escherichia coli* ATCC 25922, *Pseudomonas aeruginosa* ATCC 27853, *Staphylococcus aureus* АТСС 25923, a clinical isolate of *Pseudomonas aeruginosa* (resistant to aztreonam, ceftazidime, and cefotaxime) and *Klebsiella* spp. (resistant to tetracycline) were from the collection of the Department of Molecular Microbiology of the Institute of Experimental Medicine (Saint Petersburg, Russia). Clinical isolates of *Staphylococcus intermedius* (resistant to ciprofloxacin, cefuroxime, clindamycin, erythromycin, rifampicin, gentamicin, benzylpenicillin, and oxacillin; obtained from the infected dog bite wound) was provided by colleges from the S.M. Kirov Military Medical Academy (Saint Petersburg, Russia) and multi-drug resistant strain of *Staphylococcus aureus* 1399/17 (resistant to ampicillin, oxacillin, gentamicin, amikacin, ofloxacin, and erythromycin) by Dr. A. Afinogenova from the Research Institute of Epidemiology and Microbiology named after L. Pasteur (Saint Petersburg, Russia). The *Staphylococcus aureus* strain SG-511 was kindly given by Prof. H.-G. Sahl (University of Bonn, Germany), and the *MRSA* strain ATCC 33591 (methicillin-resistant *Staphylococcus aureus*) by Prof. R. Lehrer (University of California, Los Angeles, United States), who also supplied us with the *E. coli* ML-35р strain required for the permeabilization experiments.

#### Broth Microdilution Assay and Minimal Bactericidal Concentration Measurement

The microdilution assay was performed according to the guidelines of the European Committee for Antimicrobial Susceptibility Testing, in Müller–Hinton broth (MHB). A small aliquot of a bacterial culture, grown overnight in 2.1% MHB, was added to a fresh vial of the same sterile medium and incubated at 37°C with shaking for 2–3 h for the culture to reach log phase. The concentration of the bacterial suspension was adjusted to 1 × 10^6^ CFU/ml and mixed in equal parts with 2-fold serial dilutions of the tested substances prepared in phosphate-buffered saline (PBS). Wells containing 50% MHB/50% PBS were used to control sterility, and those containing 50% bacterial suspension in MHB and 50% sterile PBS were used to control for bacterial growth. Plates were left at 37°C for 18–20 h and the MIC then determined by visual inspection, as the concentrations that completely inhibited growth.

Samples from wells containing from MIC/2 to higher concentrations were diluted 10-fold, transferred to solid medium [3% tryptic soy broth (TSB) 1% agar] and incubated at 37°C for 18–20 h. The lowest concentration where microbial growth did not yet occur was considered the minimum bactericidal concentration (MBC). Final MIC and MBC values were determined as medians from 3 to 4 repeated evaluations.

#### Bacterial Membrane Permeability Assays

The permeabilizing effect of the tested compounds on bacterial membranes was evaluated using chromogenic markers that can be processed by bacterial enzymes when allowed to enter specific bacterial compartments. The method was first proposed by Lehrer et al. (1988) and is based on the specially modified *E. coli* ML-35p strain, which lacks lactose permeases, constitutively synthesizes cytoplasmic β-galactosidase and also produces periplasmic β-lactamase. Chromogenic substrates for these two enzymes can pass through the outer or inner bacterial membranes only if these are compromised, so their conversion to colored products provides an indication of both the kinetics and extent of membrane damage. On being given free access to the enzyme, the markers are converted to the colored products following standard enzyme kinetics. This being said, when the membrane barrier is damaged, the marker needs to cross it through the breach to reach the enzyme, so this becomes a limiting factor affecting the conversion rate, which depends on the kinetics and extent of the damage. This can be assessed by analyzing the slope of the conversion curve before it reaches its maximal level (this final concentration of the colored product depends mostly on the initial concentration of the marker). We used nitrocefin (Calbiochem-Novabiochem, United States), a substrate for periplasmic β-lactamase, as a marker of outer membrane permeability (the pink product of the reaction was monitored at 486 nm) and ONPG (*o*-nitrophenyl-β-D-galactoside, Sigma, United States), a substrate for cytoplasmic β-galactosidase, as a probe for inner membrane integrity (hydrolytic release of *o*-nitrophenyl results in a yellow color detectable at 420 nm).

*Escherichia coli* ML-35p in the stationary phase, grown overnight in 3% TSB, was washed, centrifuged at 600 *g* and 4°C for 10 min, the supernatant was removed, and the pellet resuspended in 10 mM sodium phosphate (Na-P) buffer (pH 7.4) and diluted to the concentration of 1 × 10^8^ CFU/ml. Assays were performed with 100 μl of a mixture consisting of the tested substances at 4-fold MIC, bacteria at a final concentration of 2.5 × 10^7^ CFU/ml, 10 mM Na-P buffer (pH 7.4), 100 mM NaCl and one of the chromogenic markers (20 μM of nitrocefin or 2.5 mM of ONPG). The bacterial suspension was added, and measurement began immediately thereafter. Using a SpectraMax 250 microplate spectrophotometer (Molecular Devices, United States) and SoftMax PRO software, OD data were collected every minute for 1 h at 420 or 486 nm. The test plates were kept at 37°C and shaken for 5 s before each reading. The resulting curves were plotted with Sigma Plot 11 (Systat Software Inc., United States).

#### Fluorometric Resazurin Assay for Monitoring Bacterial Metabolic Activity and Viability

Resazurin, also known by the trade name alamarBlue, has almost no fluorescence, but is converted to the pink and fluorescent resorufin by reducing agents abundantly produced by actively metabolizing cells. It has several uses that include viability testing and detection of metabolic activity in cells (Rampersad, 2012). The accumulation of this marker can be monitored both spectrophotometrically and fluorometrically, but we have chosen the latter option as the more sensitive.

The bacterial strain selected and the overall design of the experiment were almost identical to those used in the permeability tests, in order to achieve better consistency of results. The test mixtures had the same volume and practically equivalent composition, except for the replacement of the chromogenic markers with 120 μM resazurin and the addition of 0.1% MHB to provide some substrate for bacterial cells to metabolize. A control sample without bacteria was prepared to determine the baseline fluorescence value, corresponding to no metabolic activity (i.e., no viable cells).

Fluorescence intensity was measured at 590 nm, with excitation at 560 nm, every 2 min for 4 h using a Gemini EM plate spectrofluorimeter (Molecular Devices, United States) and SoftMax PRO software. 96-well flat bottom plates with opaque bottom were used for the experiment, and samples were kept at 37°C and shaken before each measurement. Collected data were plotted using Sigma Plot 11.

#### Monitoring O_2_ Consumption in Treated Bacteria Using a Clark-Type Electrode

The variation in dissolved O_2_ in the growth medium was monitored in bacterial cultures exposed to the tested compounds using an Oxigraph Plus Clark-type polarographic electrode (Hansatech Instruments, United Kingdom), assembled according to the manufacturer’s instructions. The apparatus has an electrode at the bottom of the sample chamber, from which it is separated by a selective, oxygen-permeable membrane that does not allow other components of the reaction mixture to affect the electrodes. The sample chamber itself has an adjustable volume and is thermostated by a water jacket. During the experiment, the sample is accessible through a thin channel in the upper part of the chamber, which is otherwise hermetically sealed with a rubber stopper. The assembled instrument is placed on the magnetic stirrer base, provided so that the content of the reaction vessel is constantly mixed and the electrode can react quickly to any changes that occur.

The sample mixtures for this assay had a volume of 500 μl, but otherwise their composition was the same as in the previous tests, except for the absence of the dye. After adding the bacterial suspension to it, the reaction mixture was quickly but carefully injected into the sample chamber so that no air bubbles formed, and the chamber was sealed. The volume of the reaction chamber was adjusted to the required sample volume so that no air remained in the vessel after closing the access channel. Electrode signal data, in mV, were recorded once per second using the Oxigraph Plus software provided by the manufacturer.

To translate the mV signal from the electrodes into O_2_ concentration, a two-point calibration was used, with the first reference point obtained from distilled water saturated with oxygen by using an aquarium air compressor. The signal level corresponded to the established limiting concentration of dissolved oxygen in water (Weiss, 1970) at a temperature of 37°C and the atmospheric pressure measured on the day of the experiment. The second reference point was determined using an aqueous solution of sodium dithionite (25 mg/ml), which is capable of binding and segregating free oxygen from water. The resulting signal corresponded to a zero oxygen concentration. The experimental data were plotted using Sigma Plot 11.

#### Lysoplate Assay

To verify that lysozyme conjugated to silver nanoparticles retained its enzymatic activity, we performed a standard lysoplate assay (Osserman and Lawlor, 1966). Lyophilized *Micrococcus lysodeikticus* (Sigma, United States) was added to 1% melted agarose (40–50°C) in 0.06 M Na-P buffer (pH 6.3 at RT) at a concentration of 1 mg/ml, and Petri dishes containing a 2–3 mm layer of agarose gel were prepared. Round wells with a 2 mm diameter were punched into the gel layer using a pipette with a wide tip. The tested samples were added into the wells to a final volume of 5 μl, in duplicate, as well as egg-white lysozyme (Sigma, United States) reference dilutions. Petri dishes were incubated overnight at 37°C, and the clearance zones around the wells were evaluated. Each test was repeated trice.

### Analysis of Toxic Action on Eukaryotic Cells

#### Cells Preparation and Cultivation

Peripheral blood, used to prepare erythrocyte suspensions and to extract normal neutrophils and mononuclear cells (PBMC), was obtained by qualified medical personnel from the median cubital vein of healthy volunteers and collected into vacutainer spray-coated EDTA tubes.

For hemolysis testing, whole blood was washed out twice to discard anticoagulant and plasma (by centrifuging at 300 *g* and 4°C for 10 min, removing supernatant and resuspending in ice cold PBS). The final blood pellet was considered to contain 100% erythrocytes, from which a 2.8% v/v suspension in PBS was prepared and stored at 4°C before testing for no more than 4 days.

PBMC and neutrophils were isolated from the whole blood by using density gradient centrifugation. A two-to-one PBS-blood dilution was carefully poured upon a 1.5 cm thick layer of the sterile Ficoll-400 (Pharmacia, Sweden) with a density of 1.077 and centrifuged at 600 g and 4°C for 40 min. The “PBMC ring” that remained on top of the Ficoll layer was collected with a transfer pipette, washed twice with sterile PBS and resuspended in RPMI-1640 medium (Biolot, Russia). The blood pellet forming under the Ficoll layer, containing neutrophils and erythrocytes, was additionally subjected to hemolysis in 0.83% ammonium chloride solution (~15 min incubation at 37°C) to remove residual erythrocytes, and then further processed as described for PBMC. The cells so obtained were used immediately.

K562 (human erythromyeloid leukemia) and U937 (human histiocytic lymphoma) cell lines were purchased from Biolot (Russia). A culture of normal fibroblasts was kindly offered by colleges from the Institute of Cytology RAS (Saint Petersburg, Russia). Cells were grown at 37°C and 5% CO_2_ in RPMI-1640 medium supplemented with 10% fetal bovine serum, glutamine, and a penicillin–streptomycin combination. Before toxicity testing, this medium was substituted with serum- and antibiotic-free RPMI-1640.

#### Hemolysis Test

The hemolytic activities of the compounds of interest toward human erythrocytes were assessed by measuring the amount of hemoglobin released from damaged cells. Twenty-seven microliters of the 2.8% v/v erythrocytes suspension were mixed with 3 μl of the tested compounds serially diluted in PBS to the 10 × the final test concentration. The 100% lysis control contained 3 μl of a 10% v/v Triton X-100 PBS solution instead. The 0% lysis control contained no damaging agents, only PBS. After 30 min incubation at 37°C the reaction was stopped by adding 90 μl of ice cold PBS, the samples were centrifuged for 4 min at 10,000 *g*, and 100 μl of the supernatants were transferred into a 96-well flat-bottom plate. Released hemoglobin was spectrophotometrically detected at 540 nm. The percentage of hemolysis in each sample was found as follows:


$$\text{Hemolysis}(\%)=({\text{OD}}_{\text{sample}}-{\text{OD}}_{0\%\text{lysis}})/({\text{OD}}_{100\%\text{lysis}}-{\text{OD}}_{0\%\text{lysis}})\times 100\%,$$

where OD is the optical density of the corresponding probes measured at 540 nm. Results were verified in 3–4 separate experiments, carried out in triplicate.

#### MTT-Test

Influence of the substances on the viability of normal and tumorous eukaryotic cells was evaluated using MTT [3-(4,5-Dimethylthiazol-2-yl)-2,5-diphenyltetrazolium bromide] test (Mosmann, 1983). Fibroblasts were detached from the culture flasks and transferred into 96-well sterile treated flat-bottom plates (10,000 cells per well in 90 μl) 24 h before the experiment to give them time to properly adhere. Other cells were transferred into the 96-well sterile non-treated flat-bottom plates (20,000 cells per well in 90 μl) right before the start. Ten microliters of the tested substances diluted in RPMI-1640 medium to 10 × final concentrations were added into the wells. Wells with 90 μl cell suspension and 10 μl RPMI-1640 were used as a 100% viability control; wells with 100 μl RPMI-1640 and no cells served as a 0% viability reference point. Samples were incubated at 37°C and 5% CO_2_ for 22–24 h; 4 h before the end each well was supplied with 10 μl of the 5 mg/ml MTT solution in PBS. The formation of formazan crystals (products of MTT reduction in metabolically active cells) were then dissolved by introducing 110 μl of isopropanol containing 0.04 M HCl to each well and thorough mixing. The concentration of formazan was spectrophotometrically measured at 540 nm, subtracting the unspecific background absorbance at 690 nm. The percentage of viable cells was calculated in a manner similar to the hemolysis:


$$\text{Viable cells}(\%)=({\text{OD}}_{\text{sample}}-{\text{OD}}_{0\%\text{viability}})/({\text{OD}}_{100\%\text{viability}}-{\text{OD}}_{0\%\text{viability}})\times 100\%,$$

where OD is the difference between the 540 and 690 nm absorbance values of the corresponding wells. Tests were performed in triplicates with 5–6 duplicates of each control (0 and 100%) and repeated 3–4 times.

### Ethics Statement

All blood donors gave their written informed consent before the procedure of blood collection, and the protocol was approved by the Ethical Committee of the Institute of Experimental Medicine (the Protocol 1/20 from February 27, 2020).

## Results

### Selection of AMPs for Conjugation

The AgNPs and AgNPs-antimicrobial peptide or protein conjugates investigated in this work were obtained by using the photo-reduction method, as detailed in the “Materials and Methods” section. This type of physical method for NP production has the benefit of minimizing the amount of unwanted residual components, which may alter the results of activity testing (Iravani et al., 2014; Zannella et al., 2020; Fernandez et al., 2021). The starting AgNPs were enveloped in gelatin and were the same as previously tested for synergism with AMPs and lysozyme (Zharkova et al., 2019). For the conjugates, in addition to the gelatin each incorporated one of the chosen antibacterial peptides or proteins: porcine protegrin-1 (PG-1), bovine indolicidin (Ind), hen egg-white lysozyme (Lyz), salmon protamine (Prot), or calf thymus histones (His).

These molecules were selected to have quite diverse structural and functional characteristics, which are summarized in Table 1 along with the abbreviations used for the conjugates. Some of them are already in medical use for various purposes. PG-1 and indolicidin are short and highly active, broad-spectrum AMPs, and their derivatives are among the limited number of AMPs to have reached clinical trials (Koo and Seo, 2019; Dijksteel et al., 2021). PG-1 molecules are quite rigid hairpins, due to the presence of two disulfide bonds, while indolicidin has a flexible, wedge-like structure, and they seem to act in quite different manners (see Table 1). Protamine is a longer polypeptide that substitutes somatic histones within the nucleus of sperm cells (Balhorn, 2007), and its antimicrobial properties are well-known (Aspedon and Groisman, 1996), although in medical practice it is mostly used to counter the anticoagulative effect of heparin (Boer et al., 2018). Histones are small nuclear proteins that have long been acknowledged as antimicrobial compounds alongside protamines (Miller et al., 1942). In nature, apart from their principal role in forming nucleosomes, they are thought to participate in the host defense of various epithelia and play an important role in such interesting immune mechanisms as neutrophil extracellular trap (NET) formation (Hoeksema et al., 2016). Lysozyme is one of the major antimicrobial proteins present in body fluids, secretions, and tissues of many different organisms and acts by degrading the peptidoglycan layer of susceptible bacteria (Callewaert and Michiels, 2010; Ragland and Criss, 2017). It is extensively used in food preservation and production, cosmetics, and pharmacy (Sava, 1996); for instance, in over-the-counter nasal and throat aerosols and other preparations useful against respiratory infections, as well as creams for treating topical lesions and inflammation. All these molecules therefore have promising antimicrobial properties that could add to those of the AgNPs, as long as conjugation does not mutually interfere with these properties. For this reason, we refer to them as AMPs (antimicrobial peptides, PG-1: PG-1 and Ind) or APs (antimicrobial proteins: His, Prot, and Lyz).

**Table 1:** Structure and mode of action of antimicrobial peptides and proteins used to create conjugates with silver nanoparticles (AgNPs).

Conjugate	Antimicrobial peptide or protein introduced into the coating of the nanoparticle		
	Type, name and source	Size and structure	Mode of antimicrobial action
AgNP-PG1	AMP Protegrin-1 (PG-1) porcine	18 residues (2.2 kDa) β-hairpin 2 S-S bonds; cationic (+6)^a^	Permeabilizes membranes (possibly forming toroidal pores; Sokolov et al., 1999; Lazaridis et al., 2013)
AgNP-Ind	AMP Indolicidin bovine	13 residues (1.9 kDa) linear Trp-rich; cationic (+3)^a^	Self-translocates into the cytoplasm. Acts as carrier for organic anions without forming pores (Rokitskaya et al., 2011), interferes with nucleic acids synthesis by directly binding to DNA abasic sites and inhibiting topoisomerase I (Marchand et al., 2006)
AgNP-Prot	AP Protamine salmon	32 residues (4.2 kDa) disordered Arg-rich, cationic (+2)^a^	Does not alter the overall permeability of bacterial cytoplasmic membrane or lyse cells, but affects energy transduction and amino-acid uptake, ultimately inhibiting protein synthesis (Aspedon and Groisman, 1996)
AgNP-His	AP Histones calf thymus	~ 100–220 residues (~ 11–21 kDa) globule with flexible tails Lys/Arg-rich, cationic ~ (+1.2–2.6) every 10 res.^a^	Most histones disrupt bacterial membranes. Lys-rich histone H2B can penetrate into the cells without affecting membrane integrity and bind to DNA. Fragmentation by bacterial proteases promotes activity against *E. coli*, but not *S. aureus* (Tagai et al., 2011; Morita et al., 2013; Hoeksema et al., 2016).
AgNP-Lyz	AP Lysozyme hen egg-white	129 residues (14.3 kDa) globular cationic (~ +0.6 every 10 res.)^a^	Targets the bacterial cell wall by hydrolyzing β1-4-glycosidic linkages between N-acetylmuramic acid and N-acetyl-β-glucosamine residues in peptidoglycan. An additional non-enzymatic AMP-like mechanism is proposed (membrane lysis) as catalytically inactive variants retain some antimicrobial efficacy (Laible and Germaine, 1985; Ibrahim et al., 2001; Wiesner and Vilcinskas, 2010)

a *Charge was calculated as the number of arginine and lysine residues minus the number of aspartic and glutamic acid residues (C-amidation was not taken into account)*.

### Effects of NP Conjugates on Bacterial Cells

#### Antibacterial Action

The antimicrobial activity of AMP or AP/AgNP conjugates, as well as that of the gelatin-only coated nanoparticles, was evaluated against five Gram-positive and five Gram-negative bacterial strains, including clinically isolated and antibiotic-resistant variants. Minimal inhibitory concentrations (MICs) were determined *via* the broth microdilution assay and are summarized in Table 2. The activity of the unconjugated gelatinized AgNPs seems to be fairly uniform with respect to the Gram-negative bacterial species, with a MIC of 24 μg/ml, whereas it is more varied with respect to Gram-positive bacteria, where they were found to be less effective against resistant clinical isolates. Introduction of antimicrobial peptides or proteins into the AgNP’s surface coating in most cases enhances the antibacterial potency of the resulting conjugates and broadens the activity spectrum.

**Table 2:** Antimicrobial activity of silver nanoparticle conjugates with antimicrobial peptides or proteins.

Bacteria	MIC^a^ (μg/ml)						MBC^a^ (μg/ml)
	AgNP^b^	AgNP-PG1	AgNP-Ind	AgNP-Prot	AgNP-His	AgNP-Lyz	AgNP-Lyz
Gram-positive							
*S. aureus* SG511	12	6	6	6	12	6	6
*S. aureus* ATCC 25923	12	6	12	6	24	12	12
*S. aureus* 1399/17 clin.is.	48	6^*^	12^*^	24	48	24	24
*S. intermed.* clin.is	48	6^*^	6^*^	6^*^	12^*^	24	24
*MRSA* ATCC 33591	48	6^*^	12^*^	24	48	24	24
Gram-negative							
*P. aerugin.* АТСС 27853	24	6^*^	12	6^*^	12	12	12
*P. aerugin.* clin.is.	24–48	12^~*^	12^~*^	12^~*^	24	12–24	24
*Klebsiella* spp. clin.is.	24	6^*^	12	12	12	6^*^	12
*E. coli* ATCC 25922	24	6	12	12	24	6^*^	12
*E. coli* ML-35p	24	6	12	12	24	6^*^	6
***GMIC***	***26.8***	***6.4***	***10.4***	***10.4***	***20.9***	***11.7***	—
		**PG-1**	**Indolicidin**	**Protamine**	**Histones**	**Lysozyme**	
Gram-negative							
*E. coli* ML-35p	—	6.7	10.3	10.5	76	>3,500	
*Klebsiella* spp. clin.is.	—	10	>40	40	>150	>400	
*P. aerugin.* clin.is.	—	5	10	5	38	>400	
*P. aerugin.* АТСС 27853	—	5	10	5	38	>400	
Gram-positive							
*MRSA* ATCC 33591	—	8.5	40	20	>300	>3,500	
*S. intermed.* clin.is	—	10	>40	20	>300	>400	
*S. aureus* 1399/17 clin.is	—	5	40	20	>300	>400	
*S. aureus* ATCC 25923	—	10	40	40	>300	>400	
***GMIC***	—	***7.2***	***>23.9***	***15.5***	***>138.2***	***>688.0***	

a *Minimal inhibitory concentrations (MIC) and minimal bactericidal concentrations (MBC) are the medians of 3–4 independent experiments carried out in triplicate. GMIC is the geometric mean of all the evaluated MICs*.

b *Nanoparticles coated only with gelatin*.

* *> 4-fold reduction of MIC compared with that of gelatin-only coated AgNPs*.

Conjugates containing PG-1, indolicidin, lysozyme and, to a lesser extent, protamine, show at least 4-fold reduction of the MIC against multiple bacterial species. This is in agreement with the reported antimicrobial synergism between some AMPs or APs and AgNPs^1^ (Zharkova et al., 2019) and suggests that this effect persists to some extent despite the reduced mutual mobility of the synergizing components when they are linked. Although this activity enhancement did not occur against every bacterial strain tested, the geometric mean of MIC (GMIC) values, used as overall activity criterion (Davies, 1990; Orlov et al., 2019; Wang et al., 2021), show significant improvement regarding the indicated conjugates (over 4-fold for AgNP-PG1 and over 2-fold for the others).

The mixed composition of the coating on the conjugates (AMP/gelatin) prevented the determination of the exact amount of a bound AMP or AP. A direct comparison between the MIC values of the conjugates and respective free AMP or AP is therefore not possible. However, from a comparison of the MIC values against various bacterial strains (see the bottom half of Table 2), as well as of overall GMICs, we can deduce that the effective MIC reduction is not one-sided, but applies to the AMP part of the conjugate as well as to the AgNP. This presumption also corresponds with tendencies found, for example, for chemically synthesized AgNPs conjugates with indolicidin, reported to be more effective dose-wise than both AgNPs or indolicidin alone (Zannella et al., 2020).

With respect to lysozyme, which has both an enzymatic and non-enzymatic AP capacity, we needed to check if the catalytic activity was preserved on conjugation, so we performed the lysoplate assay using the AgNP-Lyz conjugate. We also speculated that we might be able to use this method as a way of quantifying lysozyme within the conjugate, but this idea was abandoned due to the alterations in activity and substrate diffusion rate inherent to lysozyme molecules bound to a quite massive particle in a gelatin layer. Nonetheless, the method clearly demonstrated that the enzyme retains the capacity to lyse bacterial cell wall components even in the bound state (the quantitative results can be found in Supplementary Material). In this respect, it is peculiar that lysozyme enhances the activity of its AgNP conjugate more effectively against Gram-negative than against Gram-positive bacterial strains, especially considering that it has very little activity against Gram-negative bacteria in the free state.

AgNP-PG1 shows the best results among tested compounds. The least efficient, unexpectedly, was the histone conjugate, demonstrating almost no improvement compared to the unconjugated AgNPs, except for one case (see Table 2), despite showing an appreciable activity against *E. coli* and *P. aeruginosa* strains in the free state. In any case, conjugation did not cause a drop in activity.

To verify if the enhanced effect of conjugates is bactericidal in nature, or rather bacteriostatic, the MBC for one conjugate was established. AgNP-Lyz was chosen as free lysozyme has quite a poor activity against the majority of the tested bacterial strains, so its conjugate would be a likely candidate for a bacteriostatic type of mechanism. However, the MIC and MBC values coincided, or only differed by a factor of 2 (as detectable within the resolution of a two-fold dilution scheme). These data confirm that addition of AMPs or APs into the gelatin coating of AgNPs is compatible with their ability to irreversibly inactivate bacterial cells.

#### Permeabilizing Effects of AMP/AP-Conjugated AgNPs on Bacterial Membranes

To determine possible shifts in how the AMP/APs or AgNPs implement their antibacterial effects when conjugated, we first analyzed the capacity of the conjugates to permeabilize bacterial membranes, which is considered to be one of the primary mechanisms used by AMPs to kill bacteria.

**Figure 1:**
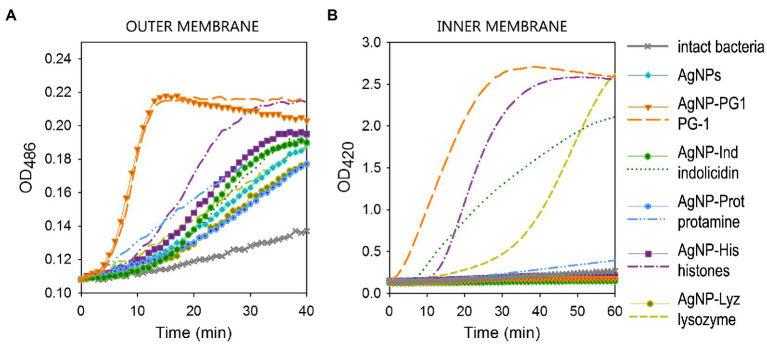
Permeabilizing effect of AgNP-AMP/AP conjugates on *Escherichia coli* ML-35p outer **(A)** and inner **(B)** membranes. The effects of the conjugates are compared to those of the corresponding antimicrobial peptides and proteins (AMPs and APs) and gelatin-only coated silver nanoparticles (AgNPs) alone. The optical density (OD) increase correlates to the hydrolysis of the chromogenic markers by bacterial enzymes. Permeabilization of the outer membrane gives the nitrocefin marker the access to periplasmic β-lactamase, while inner membrane permeabilization gives the *o*-nitrophenyl-β-*D*-galactoside marker (ONPG) the access to cytoplasmic β-galactosidase. Bacterial membranes are impenetrable to the markers under normal conditions; hence the dynamics of their degradation allow assessing the scale and velocity of membrane damage inflicted by the tested substances. The concentration of antimicrobials used was equal to 4 × MIC (minimal inhibitory concentration); typical curves are shown.

Figure 1A clearly indicates that all the tested conjugates, as well as AgNPs coated only in gelatin and non-conjugated polypeptides, to some extent increased the permeability of the outer membrane in *E. coli* ML-35 with respect to untreated bacteria, albeit with different kinetics. Worthy of note is the remarkable similarity between the curves for the highly lytic PG-1 in the free and conjugated form, including the characteristic sharp rise and abrupt transition to a descending plateau that distinguishes the behavior of the free AMP. This indirectly confirms that PG-1 retains its specific activity within the conjugate. Histones and protamine do loose some permeabilizing capacity in the conjugated state, but the conjugates show a permeabilizing capacity that is slightly better or comparable to the non-conjugated AgNP.

Contrary to the observations on the outer membrane permeability, the effect of AgNP-AMP/AP conjugates on the cytoplasmic bacterial membrane of *E. coli* ML-35 is markedly reduced with respect to the corresponding free polypeptides (Figure 1B). The extent and kinetics of permeabilization for the free polypeptides ranges from rapid and extensive to slow and weak, but all demonstrated an appreciable effect on the cytoplasmic membrane during the 1 h period of contact with bacterial cell. The only exception was protamine, which is known to have a non-permeabilizing interaction with bacterial plasmalemma (Aspedon and Groisman, 1996; Pink et al., 2014). None of the conjugates, however, caused a significant increase in the inner membrane permeability to ONPG, acting in a similar manner to the gelatin-only coated AgNPs.

#### Dynamics of the Inhibition of Bacterial Cells Metabolism

An active living cell is characterized by a high level of metabolic processes, so that the metabolic rate is a fairly accurate indicator of its viability. Bacterial cells are no exception. Faced with unfavorable environmental conditions, bactericidal agents included, bacteria can initially intensify their metabolism by mobilizing available resources to activate “fight-or-flight” strategies to counter the negative effect. The sufficiently prolonged and/or potent impact, however, inevitably results in the suppression of both anabolism and catabolism. In this sense, assessing the metabolic activity of a bacterial culture can provide information about time-kill dynamics.

To evaluate the impact of conjugated nanoparticles on bacterial metabolism, we used a fluorometric method based on the redox-sensitive dye resazurin. The bacterial respiratory chain is considered to be the major supplier of reducing agents for resazurin, so its reduction is often interpreted as a specific measure of aerobic respiration. Apart from its use as a marker for overall metabolic activity, it should also be considered that the membrane located respiratory machinery is presumably a major focus for the detrimental action of both nanoparticulate and ionic forms of silver.

**Figure 2:**
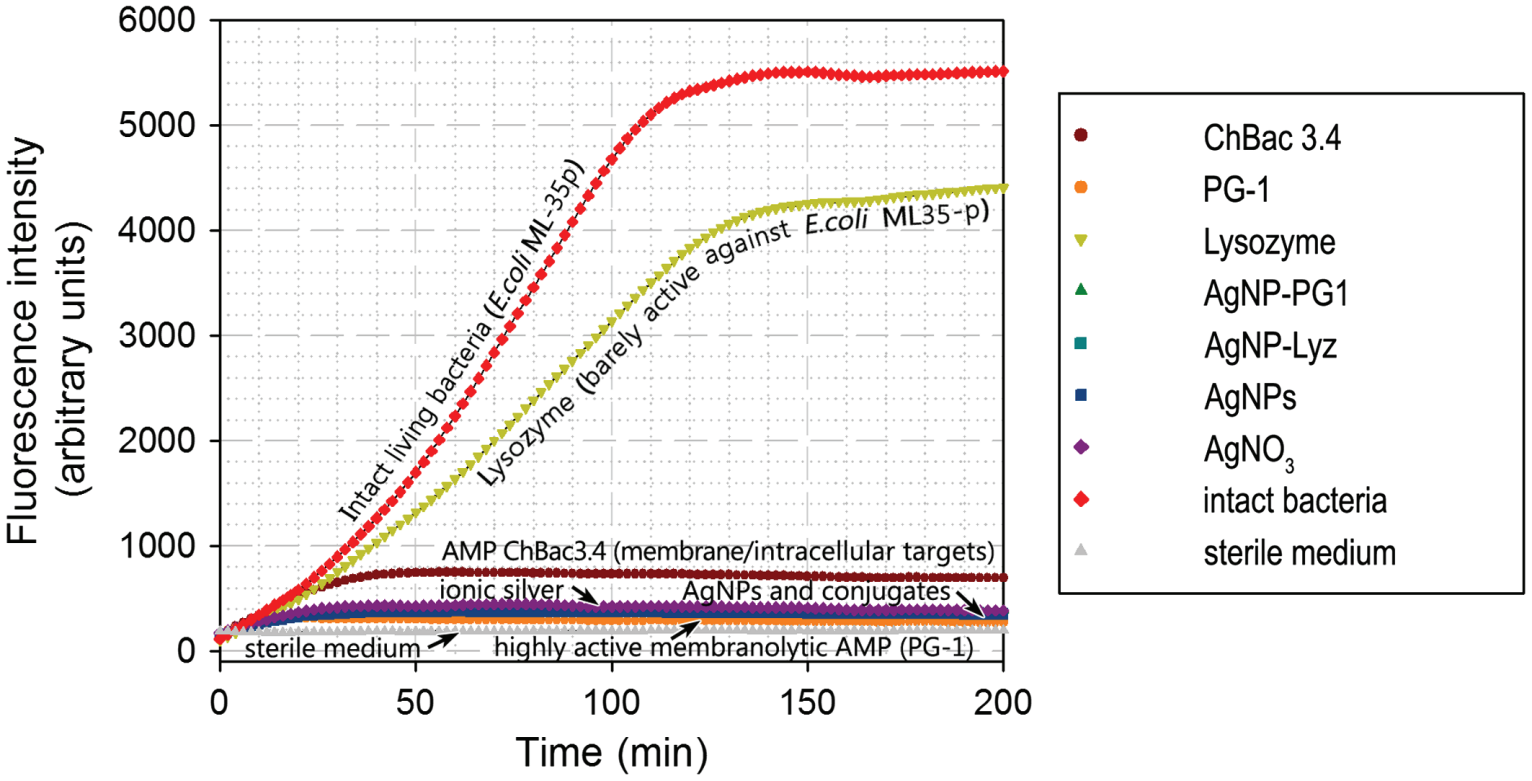
Dynamics of *E. coli* ML-35p metabolic activity inhibition by AgNP-AMP/AP conjugates. Effects were compared to those of antimicrobial peptides and proteins (AMPs and APs), gelatin-only coated silver nanoparticles (AgNPs) and ionic silver (AgNO_3_). An actively metabolizing cell supplies the redox-sensitive marker resazurin with metabolic reducing agents, turning it into the fluorescent resorufin. An increase in fluorescence intensity thus indicates actively metabolizing bacteria. The tested concentration of the antimicrobials was equal to 4 × MIC (minimal inhibitory concentration); typical curves are shown.

The impact of AgNP-PG1 and AgNP-Lyz conjugates on the metabolic activity of *E. coli* ML-35p was compared with the effects of their components – gelatinized AgNPs and free PG-1 or lysozyme. For comparison, we also tested the action of ionic silver, in the form of silver nitrate, and of an AMP, ChBac3.4, that uses both a membranolytic and an intracellular mechanism to kill bacteria (Shamova et al., 2009; Kopeikin et al., 2020; Figure 2). The bacterial species, growth phase, tested compound concentrations and overall conditions were the same as used in the membrane permeability assay.

Under these conditions, lysozyme, which is known to be poorly active against *E. coli* (see also Table 2), had only a minor effect on the metabolic state of the bacterium. On the other hand, the partly membranolytic ChBac3.4 had a significant dampening effect on respiration and the highly lytic PG-1 completely abrogated it. Ionic silver, gelatin coated AgNPs and their conjugates with antimicrobial peptides and proteins also had this effect, and it unfolds rapidly. The divergence from the positive control (intact living bacteria) is noticeable after just 10 min from the start of incubation, and resorufin fluorescence (the product of resazurin reduction) reaches a plateau after about 30 min, suggesting the full inhibition of bacterial respiration and metabolism.

These results support the idea that both the AgNPs and their conjugates rapidly impede the correct functioning of the bacterial respiratory chain. However, the fact that the highly lytic PG-1 on its own has a similar effect to gelatinized or conjugated AgNPs makes it difficult to say if the respiratory chain is directly targeted by the tested antibacterial agents, or is simply due to rapid collateral damage to the membrane surrounding the respiratory protein machinery and/or to ion leakage and subsequent dissipation of the transmembrane potential. However, taking into account that the less lytic ChBac3.4 has a smaller effect on respiration, and that permeabilization experiments did not indicate a strong disruptive effect on the cytoplasmic membrane by AgNPs or their conjugates, the direct action of the latter on the bacterial respiration seems a more probable explanation.

#### Dynamics of the Inhibition of O_2_ Consumption by Bacteria

The potential effects on bacterial aerobic respiration was further investigated from a different angle, namely by monitoring the consumption of O_2_ dissolved in the growth medium by bacterial cells in the presence or absence of the antimicrobial compounds of interest.

**Figure 3:**
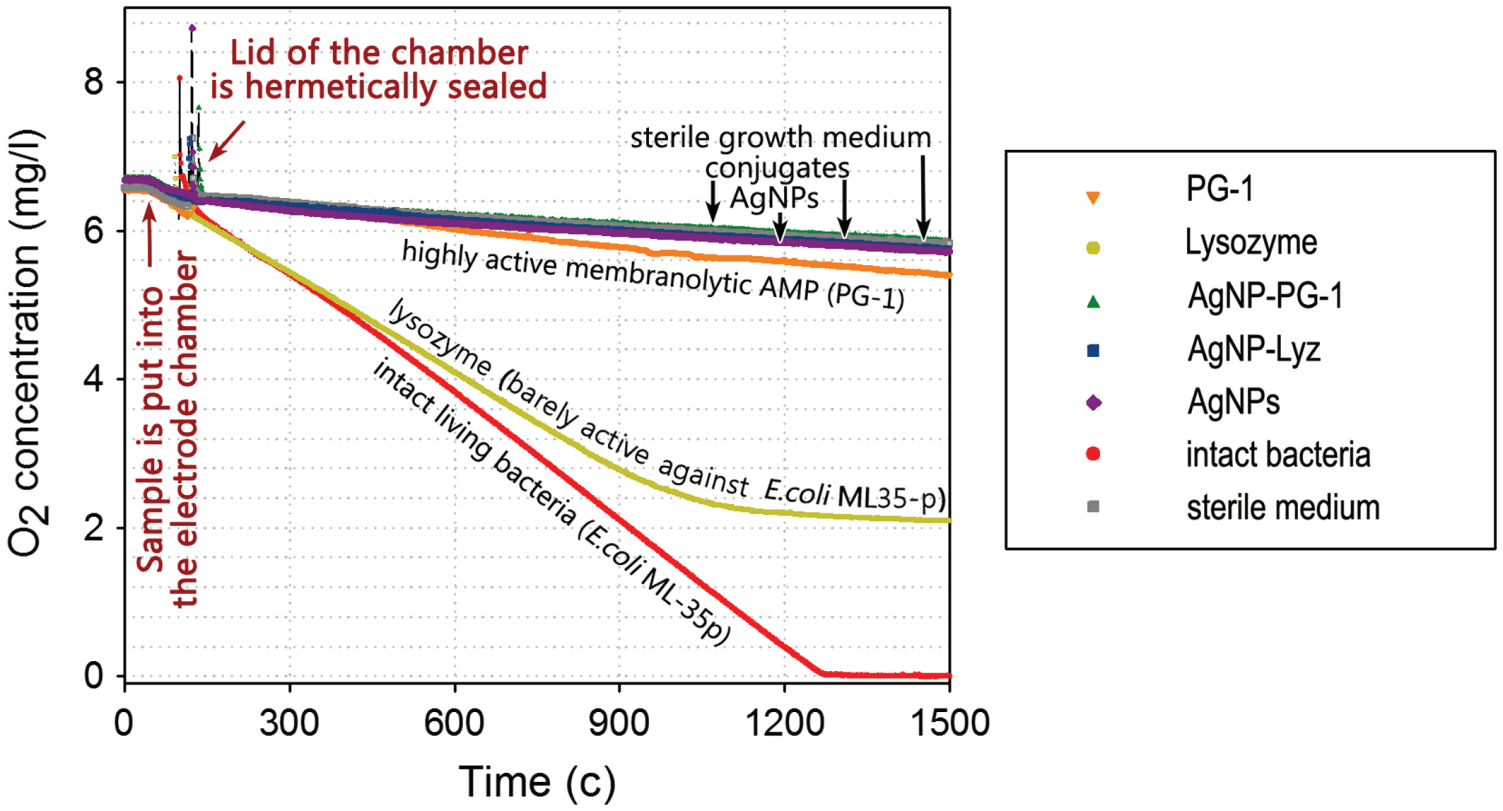
Dynamics of the consumption of O_2_ dissolved in the growth medium by *E. coli* ML-35p. Molecular oxygen consumption was measured in the presence of AgNP-AMP/AP conjugates or antimicrobial peptides and proteins (AMPs and APs) and gelatin-only coated silver nanoparticles (AgNPs) alone. The concentration of dissolved oxygen was measured using an Oxigraph Plus Clark-type electrode unit with hermetically sealed lid (Hansatech Instruments ltd, UK). The concentration of antimicrobials in the test mixture was equal to 4 × MIC (minimal inhibitory concentration); typical curves are shown.

We maintained the testing conditions as far as possible to the previous assays to allow comparison of the experimental data, and with the same bacterium (*E. coli* ML-35), save for the use of a specialized electrode chamber with a hermetic lid and a liquid thermostat instead of a 96-well plate, affecting the final volume. The dissolved O_2_ concentration in the liquid medium, measured using Clark-type electrode over time in a hermetically sealed test chamber containing experimental mixtures with a suspension of *E. coli* ML-35p cells (see “Materials and Methods” section) is shown in Figure 3.

As can be seen, intact bacteria actively consume oxygen at an almost constant rate so the O_2_ concentration within the electrode unit decreases linearly to a value close to zero, in about 20 min. *E. coli* exposed to lysozyme, to which it is relatively resistant (see Table 2), also allows a significant decrease in the oxygen concentration, although consumption eventually ceases. The membranolytic peptide PG-1 instead effectively inhibits oxygen consumption, with only an initial slight drop in dissolved O_2_ (~6% of the total) in first 20–25 min, compared to negative control (without any breathing bacteria).

AgNPs, regardless of the presence or absence of PG-1 or Lyz in the gelatin coating, effectively blocks oxygen consumption. Unlike the cellular metabolism assay, this experiment can therefore detect a small difference in the effect of AgNPs and the highly membranolytic PG-1, and the former are more potent. These data further support the assumption that bacterial respiration is specifically targeted by AgNPs as well as by their conjugates with AMPs and APs, and that its disruption plays an important role in ensuring the bactericidal effect of these nanoparticles.

### Effects Toward Eukaryotic Cells

#### Hemolytic Action

It is well-known that mature mammalian erythrocytes do not contain intracellular organelles such as nucleus, mitochondria or ribosomes (Moras et al., 2017), which are common targets for cytotoxic molecules. Thus, red blood cells (RBCs) are considered to be a convenient model to specifically test membrane-oriented damaging effects of various compounds, despite the fact that RBC membranes have some peculiarities with respect to other eukaryotic cells.

**Figure 4:**
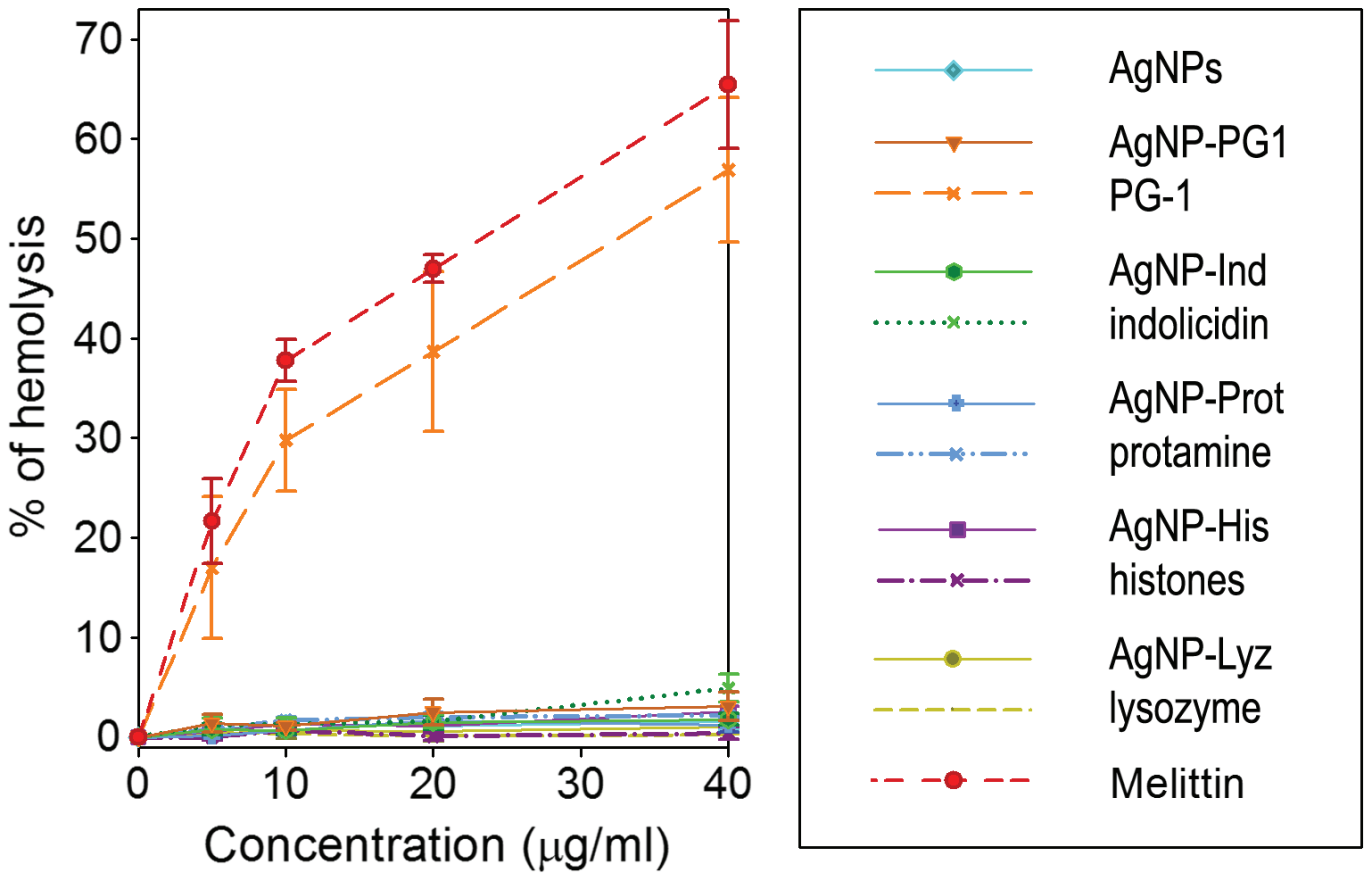
Hemolytic activity of AgNP-AMP/AP conjugates. Hemolysis was compared to that caused by the corresponding antimicrobial peptides and proteins (AMPs and APs) and gelatin-only coated silver nanoparticles (AgNPs) alone. Results are shown as mean ± standard deviation and are calculated based on 3–4 independent experiments performed in triplicate. Well-known hemolytic peptide melittin (Sigma) from the honeybee venom is used as a reference hemolytic compound.

We investigated hemolytic action of the AgNPs and their conjugates with AMPs and APs and compared them to the unbound molecules known to be significantly hemolytic. The effect was observed at similar concentrations to the microbicidal ones shown toward the majority of the examined strains of microorganisms. As can be seen in Figure 4, all conjugates, as well as gelatin-only coated AgNPs within the tested range of concentrations do not cause significant hemolysis of human erythrocytes. Hemolysis is very low up to the highest tested concentration, suggesting that, limited hemolycity can be expected even well above 40 μg/ml, the maximum tested concentration. This is also the case for the AgNP-PG1 conjugate, even though the free peptide has HC_50_ below 40 μg/ml and shows significant hemolycity at even the lowest tested concertation (~20% at 5 μg/ml). Thus, conjugation of membranolytic AMPs with nanoparticles may be an effective method of reducing their hemolytic activity and consequent toxicity to the host.

#### Cytotoxic Effects Toward Normal and Tumor Cells

**Figure 5:**
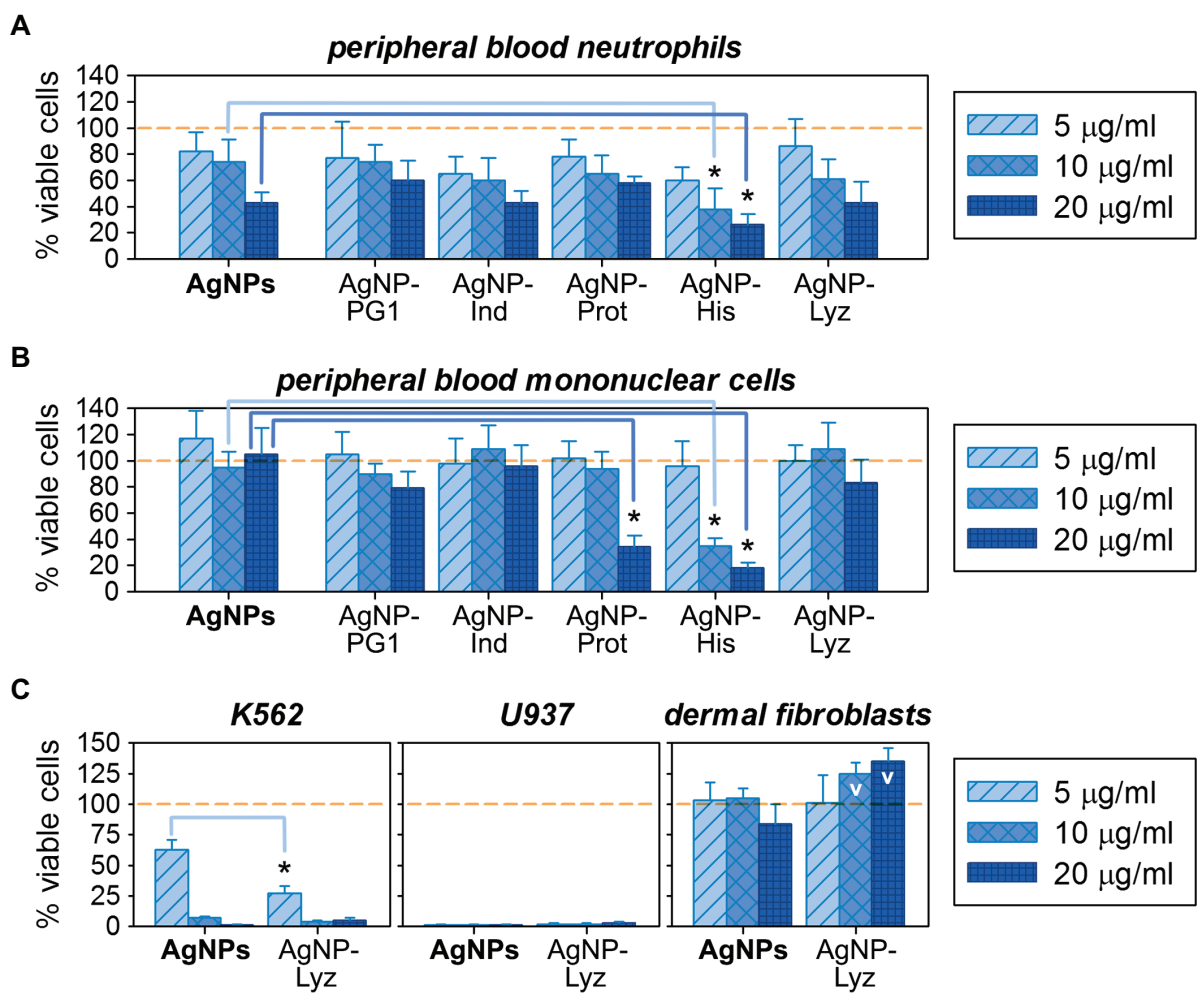
Comparative cytotoxic activity of gelatin-only coated silver nanoparticles (AgNPs) and their conjugates with antimicrobial peptides and proteins (AMPs) on eukaryotic cells: on normal human peripheral blood neutrophils **(A)** and mononuclear cells **(B)**; on human cancer cell lines K562 and U937, as well as on normal human dermal fibroblasts **(C)**. Mean ± standard deviation values were calculated based on 3–4 independent MTT-tests performed in triplicate. Cases of significantly increased toxicity compared to the same concentration of unconjugated AgNPs are marked with asterisks (Mann–Whitney U-test, *p* < 0.05); cases of statistically significant increase of the presumed viable cell number over the non-treated control (100% viable cells) are denoted with ^V^ (Mann–Whitney U-test, p < 0.05).

Regarding cytotoxicity, cell lysis is obviously not a necessary or exclusive scenario for toxic action. According to the literature, the toxicity of AgNPs against eukaryotic cells is mediated, among other things, *via* their adverse effects on DNA and mitochondrial functioning (Durán et al., 2010). The toxic activity of AMPs is also not limited to disruption of the plasma membrane – other membranous organelles as well as negatively charged molecules (including nucleic acids) could be affected by them (Deslouches and Di, 2017; Tornesello et al., 2020). For this reason, analysis of the cytotoxic effects of AgNP-AMP/AP conjugates was extended to mammalian cells containing the full repertoire of organelles: human mononuclear cells and neutrophils obtained from the peripheral blood of healthy donors, as summarized in Figure 5A,B.

The % of viable cells after 18–24 h incubation with the tested compounds was evaluated using the MTT assay in a set of 3–4 independent experiments, and effects of AgNP-AMP/AP conjugates were compared to gelatinized AgNPs at the same concentrations. This allows assessing any influence that the AMP or AP in their outer coatings has on the overall toxicity of the conjugates. Though not marked, the shift in activity between AgNPs and their AMP/AP conjugates are somewhat more prominent then in hemolysis test. At low concentrations, a relatively minor toxic effect on normal human white blood cells is observed, being more pronounced toward neutrophilic granulocytes. In general, under these conditions, the viability of eukaryotic cells exposed to AgNP-AMP/AP conjugates is similar to unconjugated AgNPs. Toxicity seems to increase at higher concentrations, but only the AgNP-His conjugate shows a statistically significant increase in toxicity with respect to the unconjugated nanoparticles. Despite the acknowledged extracellular role of histones in immune processes such as NET formations, due to their predominant nuclear localization extracellular release is usually taken as a damage-associated molecular pattern (DAMP; Chen et al., 2014). The specific signaling effects of extracellular histones, such as the ability to directly trigger NETosis (Abrams et al., 2013), may indeed contribute to the observed toxicity of the AgNPs-His conjugate. Interestingly, the toxic effect relative to unconjugated AgNPs is even more prominent for mononuclear cells, which are also susceptible to the AgNP-Prot conjugate (also a nuclear protein). Whether the same underlying mechanism is involved requires verification.

**Figure 6:**
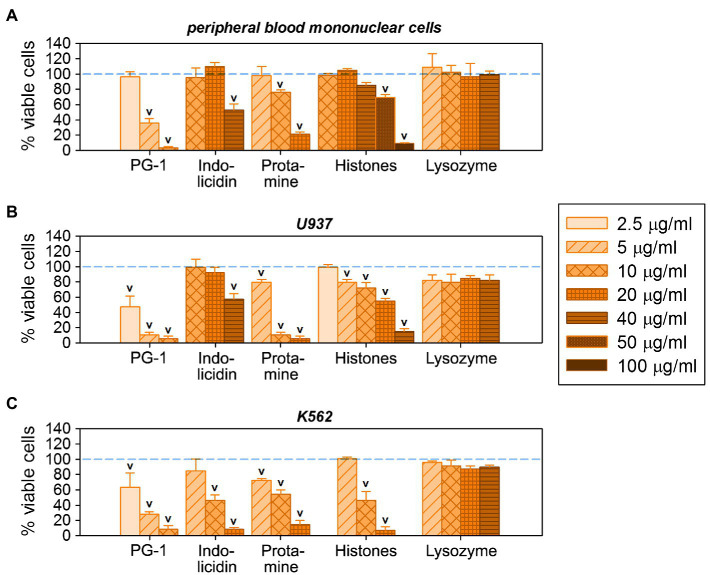
Individual cytotoxic activity of antimicrobial peptides and proteins (AMPs and APs) used for conjugation on eukaryotic cells: on normal human peripheral blood mononuclear cells **(A)**, as well as on human cancer cell lines U937 **(B)** and K562 **(C)**. Mean ± standard deviation values were calculated based on three independent MTT-tests performed in triplicate. Cases of statistically significant toxic effect in comparison with non-treated control (100% viable cells) are denoted with ^V^ (Mann–Whitney U-test, p < 0.05).

The AgNP-Lyz conjugate seems promising, considering the antimicrobial activity and overall low toxicity toward normal eukaryotic cells. It was therefore additionally evaluated against two tumor cell lines [K562 (human erythromyeloid leukemia) and U937 (human histiocytic lymphoma)], as well as against normal human skin fibroblasts. The effect of AgNP-Lyz compared to unconjugated AgNPs is shown in Figure 5C. Both conjugated and unconjugated AgNPs have a pronounced and concentration dependent cytotoxic effect on both lines of tumor cells, in contrast to the mild toxicity observed for normal white blood cells. In fact, even the lowest chosen concentration inactivated 100% of the U937 cell line. For the K562 cell line, the addition of lysozyme (which is not known for a direct antitumor activity by itself, unlike some other AMPs and APs, see Figure 6B,C) to the nanoparticle significantly enhanced potency at the lower concentrations.

Dermal fibroblasts are usually quite resistant to the toxic compounds, as also observed here. In fact, a dose-dependent increase in % viable cells seems to occur in the presence of AgNP-Lyz conjugate, which was not present for unconjugated AgNPs. However, as the MTT-test is based on the intensity of cellular metabolism, one cannot unquestionably translate the results to an increased cell count, it might be due to a more intense metabolic rate. Nevertheless, the observed trend suggests that the presence of lysozyme on the nanoparticles mediates some specific effect on metabolism, that is possibly growth-stimulating or proliferative, which is absent in unconjugated AgNPs.

Comparing the cytotoxicity of AgNP-AMP/AP conjugates with those of the corresponding unbound AMPs and APs, as summarized in Figure 6, one may perceive the same trend of effectively lowering the nonspecific damage by highly membrane-active peptides such as PG-1 toward normal cells as that found in the hemolysis assay. *Vice-versa*, for those AMPs or APs whose toxicity is already lower than that of the gelatin-only coated AgNPs, these do not further decrease the cytotoxicity of the latter. Considering the activity of histones towards mononuclear cells (Figure 6A), some presumably specific effects can even intensify after the conjugation.

## Discussion

Nanoparticles, at an intermediate scale to molecules and large biological structures, such as cells and organelles, provide an attractive platform for drug design. They have a sufficiently large surface area to provide ample opportunities for the creation of multimolecular and thus multifunctional complexes, while at the same time exhibiting good penetration potential. In any case, their functionalization is a necessity for improved stability, both as a powder or in stock solution and in the biological environment. On entry into an organism, nanoparticles rapidly attract biomolecules (mostly proteins) that dwell on their surface, forming a rather thick “corona” that can ultimately interfere with their intended function or, if formed by opsonins, promote clearance by macrophages (Chonn et al., 1992; Tenzer et al., 2013; Gao and He, 2014; Chen et al., 2017). However, exploiting an “if you cannot beat them, join them” approach, i.e., designing nanoparticles that are already surface-conjugated to peptides or proteins and where the composition of the coating is predetermined and controlled, is considered as a promising and versatile strategy to solve this problem and develop effective and highly biocompatible nanodrugs (Spicer et al., 2018). It can have a reciprocally beneficial effect on the molecules introduced into the coating, for example, solving bioavailability and/or stability issues for protein and peptide therapeutics. The fact that multimolecular complexes can be designed provides a very flexible toolkit for tuning selectivity, activity spectrum and other properties of the final product.

In this work, we have focused primarily on the prospect of synergizing the intrinsic antimicrobial activity of AgNPs with that of antimicrobial peptides (AMPs) and proteins (APs) by entrapping them within the gelatinized coating of the nanoparticles. Since there already was evidence that silver nanoparticles and AMPs can enhance each other’s antibacterial potency, we speculated that this would also be the case when they were conjugated. Combination therapy is a useful tool to combat bacterial resistance, as it not only increases activity against already drug-resistant pathogens, but also presumably slows the selection of resistant variants among exposed bacteria (Zimmerman et al., 2007; Turnidge, 2014; Dijksteel et al., 2021). The synergy between antimicrobial compounds used in combination provides an additional opportunity to minimize dose and associated side effects (Chou, 2006). This would be advantageous in the case of AgNPs and AMPs combinations, as both are known to exert some cytotoxic effects on animal cells. Thus, chemical conjugation of such agents may prove a useful option for converging combinational therapy with useful chemical modifications and delivery approaches.

With respect to synergy, on the one hand the binding of the two components into a stable complex co-delivers them to the site of action at high local concentrations, which should help manifest it. On the other hand, the formation of conjugates could limit the mobility of AMPs or APs and could lead to conformational changes or steric hindrances that affect their capacity to meet up with microbial cell targets, compromising their biological action, which would offset it. To assess the effects of conjugation on antimicrobial synergy, as well as its influence on the mode of action of the conjugated components, we have analyzed the complexes of AgNPs with five different types of AMPs and APs. These were selected to differ in length, secondary structure, and antimicrobial mechanism (as summarized in Table 1), allowing us to weigh how these factors contribute to the outcome.

Comparison of the intrinsic antimicrobial activity of unconjugated, gelatinized AgNPs (the core structure) with that of conjugates linked to AMPs or APs capable of exerting their own specific type of antimicrobial activity, confirmed that synergy can be preserved in the conjugated state. Most conjugates demonstrated a significant reduction in MIC values with respect to unconjugated nanoparticles (4-fold or more in the case of certain bacteria, resulting in a 2–4 fold improvement of overall GMICs) and were also more active than corresponding unbound AMPs or APs alone. These probably profit from the local concentration increase at the nanoparticle surface. Furthermore, conjugates with PG-1 and indolicidin maintained a high activity, indicating that such factors as the small size and/or a rigid structure of these peptides does not prevent them from retaining a sufficient antimicrobial efficacy upon binding to AgNPs.

Literature data seems to indicate a greater activity of AgNPs against Gram-negative bacterial species than Gram-positive ones (Shrivastava et al., 2007; Mukunthan et al., 2011; Dehnavi et al., 2013; Yun’an Qing et al., 2018). This leads to the consideration that bacterial susceptibility to AgNPs depends, among other factors, on the thickness of the peptidoglycan layer, which is supported by some (Li et al., 2005), but not other (Vazquez-Muñoz et al., 2019), reports on the synergy between AgNPs and β-lactam antibiotics. We, however, found that the antimicrobial effectiveness of nanoparticles correlated more with the resistance profile of bacteria than with their nature. This may be an indication that some of the acquired resistance mechanisms employed by multiresistant bacterial strains against conventional antibiotics are effective also against silver-based compounds. Such strategies, which often depend on preventing penetration into the cell, include loss of outer membrane porins or expression of efflux systems (Li et al., 1997; Randall et al., 2015; McNeilly et al., 2021). Alternatively, they could involve sequestration of the antimicrobial agent in the vicinity of the cell, for example by extracellular polymeric substances (EPS) within the biofilm (Kang et al., 2013; Panáček et al., 2018; McNeilly et al., 2021).

It is tempting to speculate that the nonspecific increase in bacterial membrane permeability caused by membranolytic AMPs could somehow help to overcome some of these mechanisms. The fact, that PG-1, the most membranolytic of the tested AMPs, increases the antimicrobial activity of the corresponding AgNP-PG1 conjugate most efficiently, is in agreement with this assumption. However, the conjugation of all the AMPs or APs to the nanoparticle seemed to abrogate the ability to permeabilize the inner membrane of *E. coli*. A significant capacity to permeabilize the outer membrane was maintained by the conjugated nanoparticles, but histones, which were second only to PG-1 in this, did not improve the activity of conjugated AgNPs with respect to the unconjugated ones. The role of conjugation on the capacity of AgNPs to reach the bacterial surface or to mediate other aspects of their antimicrobial activity needs further study. In this respect, the fact that lysozyme, which is selectively more active toward Gram-positive bacteria, upon conjugation predominantly enhances the antimicrobial potency of the nanoparticle toward Gram-negative strains, is in itself interesting. It suggests that its effect in the conjugate is not related to its hydrolysis of peptidoglycan, thus facilitating penetration of the NP to the membrane surface, and this is consistent with data reported by other researchers, who found that AgNPs coated with heat-denatured lysozyme are intriguingly more bactericidal than those coated with natural enzyme (Ashraf et al., 2014).

The fact that membrane permeability assay, while confirming this capacity for unbound AMPs and APs, showed no permeabilization by conjugated AgNPs, is in a way consistent with literature data that suggests large silver nanoparticles remain on the surface of bacterial cells (Sánchez-López et al., 2020). However, one must take into account the fact that the method used relies on exposing cytoplasmic β-galactosidase to its substrate, and it is well-known that silver ions, among other effects, disrupt the functioning of cellular enzymes (Dakal et al., 2016; McNeilly et al., 2021). We have in fact found that β-galactosidase mediated cleavage of the chromogenic marker ONPG, used as a permeability indicator, can be seriously affected by silver ions but that this inhibition does not occur instantly, so that an initial small signal is in any case detected. A rapid and pronounced membranolytic effect by the AgNPs should therefore still be detectable, and this was not the case even for AgNP-PG1. This was further supported by the hemolysis assay, which does not depend on an enzymatic activity, and where a low capacity to permeabilize RBC was observed for all conjugates, including the one with the quite hemolytic PG-1.

According to existing models, the formation of pores or lesions in the membrane requires the cooperative action of several AMPs molecules, and it can be assumed that binding to a nanoparticle prevents these molecules from assembling for this purpose. Even if these should form, passage of the bulky conjugated nanoparticle through them is unlikely and the nanoparticle itself may serve as a stopper. Admittedly, if bound AMPs at least preserve some capacity to disturb the membrane, this could still allow small ions such as released Ag^+^ into the cell. The fact that no permeability was detected for the larger chromogenic marker ONPG does not negate this possibility.

With respect to the mode of antibacterial action of AgNP-AMP/AP conjugates, the effect on bacterial aerobic metabolism and O_2_ consumption was most revealing, as an alternative to membrane disruption. We observed that a rapid and intensive inhibition of these processes occurs in the presence of AgNPs, irrespective of conjugation, which tallies with the bactericidal nature of their action, and is supported by similar MIC and MBC values. The inhibition was consistent across the conjugated AgNPs, suggesting that it is mediated principally by the AgNP core, and markedly higher than that caused by PG-1, the most membrane disruptive of the studied peptides and proteins, on its own.

It is known that AMPs, especially membrane-active ones, affect the transmembrane potential by allowing various ions to passively diffuse through bacterial membranes. This potential is an essential component of the proton motive force alongside the proton concentration gradient; hence, its diminishing has a rapid and drastic effect on the functioning of bacterial cell respiration, and cuts short the energy supply, resulting in the overall inhibition of bacterial metabolism. Bolintineanu et al. (2010) have demonstrated that under conditions quite similar to the ones we used while assessing metabolism inhibition and decrease in O_2_ consumption by PG-1 [10^8^ CFU/ml *E. coli* ML-35p in the presence of 100 mM NaCl, 10 mM sodium phosphate (pH 7.4) and 25 μg/ml peptide] the induced K^+^ release reaches up to the 80% level within the first 2 min, resulting in a 100% transmembrane potential decay in 6–8 min. This corresponds well with the time frames for inhibition of bacterial metabolic activity and O_2_ consumption we found. The fact that AgNPs and their AMP/AP conjugates cause an even more abrupt stop in oxygen uptake suggests that they may act in a more direct or multifactor manner on the bacterial respiratory chain than any of the unconjugated polypeptides, including highly membranolytic PG-1.

Unfortunately, the effect is so fast that it masks possible AMP/AP-specific contributions to the rate of ion leakage and drop in membrane potential, which could add some insight on the causes underlying the observed activity differences (i.e., different MIC values) between conjugates and gelatin-only coated AgNPs.

Regarding the cytotoxicity of the silver nanoparticle conjugates, as mentioned above, hemolysis assays showed this to be low and not dependent on the hemolytic properties of the conjugated polypeptides. However, analysis of the effects on other types of eukaryotic cells revealed some interesting AMP/AP-mediated features in the action of the tested compounds. For instance, the AgNP-His conjugate, and to a lesser extent also AgNP-Prot, showed increased toxicity to normal neutrophils and mononuclear cells, which in a way demonstrates that conjugated AMPs or APs at least in part retain their capacity to interact with these cells even when conjugated. This may allow them to continue to express useful immunomodulatory properties at nontoxic concentrations. Also of interest is the positive effect observed for the AgNP-Lyz conjugate on human skin fibroblasts, which seems to enhance their metabolism and possibly promote cellular proliferation, in agreement with the previously reported stimulatory proliferative effects of lysozyme on fibroblasts (Guo et al., 2007; Cao et al., 2012).

A striking selectivity was observed for the cytotoxic effect of conjugates toward normal and tumor white blood cells, raising the prospect of using AgNPs or their AMP/AP conjugates as antitumor agents. This is particularly the case for the AgNP-Lyz conjugate. Admittedly, the antitumor potential of lysozyme on its own has not been fully elucidated (in the context of simple *in vitro* assays). Its activity *in vivo* may be mediated by immunostimulatory effects (Sava et al., 1989), and a more direct involvement in the regulation of epithelial cell proliferation, which has also been shown on gastric cancer cells, is not necessarily associated with an actual decrease of cellular viability (Guo et al., 2007). In our hands, statistically relevant effects of free lysozyme on the K562 cell-line required at least a 10-fold higher concentration than the highest tested concentration of AgNP-Lyz, in an MTT-assay (Zharkova et al., 2019). We can therefore assume that the observed potentiation of antitumor activity is caused by a nonspecific enhancement of adhesion to tumor cells. A similar phenomenon caused by a positively charged polymeric coating is well-known in relation to antimicrobial activity (El Badawy et al., 2011; Tang and Zheng, 2018). Strictly speaking, the observed similarities in the action of different conjugates argues for such a purely electrostatic effect playing an important role in the antimicrobial and antitumor potential of the nanoparticles likewise, in both cases exploiting the anionic nature of the target cell surfaces. On the other hand, some of the assays used confirmed that the antimicrobial peptides and proteins introduced into the AgNP envelope retain some of their biological effects, at least those mediated by interaction with the surface structures of bacterial or eukaryotic cells.

In summary, conjugation of AgNPs with antimicrobial peptides or proteins offers a number of advantageous possibilities. In the first place, it can efficiently reduce the undesirable collateral toxicity of membranolytic AMPs toward normal host cells. Moreover, highly membranolytic AMPs may have the best prospects of amplifying the AgNPs’ antimicrobial activity and potential to successfully combat drug-resistant pathogens. In the second place, apart from directly enhancing antimicrobial efficacy, AMP/AP-based coating allows functionalization toward other beneficial properties, such as immunomodulatory and wound healing effects. As materials containing silver nanoparticles are increasingly used in wound dressings, such a possibility is interesting. However, our data also highlight that the development of nanoparticle/AMP or /AP conjugates should be approached with caution, taking into account a variety of other possible *in vivo* effects that need to be investigated before their potential can be fully assessed. Not all of AMP- or AP-mediated responses may be advantageous; for example, some of these compounds may promote opsonization, which would negate their other beneficial properties.

## Data Availability Statement

The original contributions presented in the study are included in the article/Supplementary Material; further inquiries can be directed to the corresponding author.

## Author Contributions

OS, OG, and AT contributed to the conception and design of the study, data analysis, and writing of the manuscript. MZ wrote the first draft of the manuscript and performed all antimicrobial testing experiments, evaluation of the effects of AMP/nanoparticles complexes on bacterial membrane permeabilization, MTT tests, and the statistical analysis of the obtained data. OG synthesized and characterized silver nanoparticles. AD contributed to the conception and design of the study and performed the fluorometric resazurin assay for monitoring bacterial metabolic activity and viability. DO was responsible for the experiments on the monitoring O_2_ consumption by bacteria with polarographic Clark-type electrode unit. EV carried out experiments on the examination of the hemolytic activity of the studied samples. All authors contributed to the article and approved the submitted version.

## Funding

This work was carried out with the financial support of the Ministry of Science and Higher Education of the Russian Federation, Agreement N° 075-15-2020-902 (World-Class Research Center “Center for Personalized Medicine”).

## Conflict of Interest

The authors declare that the research was conducted in the absence of any commercial or financial relationships that could be construed as a potential conflict of interest.

## Publisher’s Note

All claims expressed in this article are solely those of the authors and do not necessarily represent those of their affiliated organizations, or those of the publisher, the editors and the reviewers. Any product that may be evaluated in this article, or claim that may be made by its manufacturer, is not guaranteed or endorsed by the publisher.

## Footnotes

^1^The reported synergy was assessed using checkerboard titration method and calculation of the fractional inhibitory concentration indices FICI = [A]/[MIC A] + [B]/[MIC B], where [A] and [B] are the concentrations of substances A and B in their effective combination. Synergy was indicated by FICI ≤ 0.5, which, considering the method applied, required an at least 4-fold reduction of the concentration of both components compared to their individual MICs.
