# Supplementary material for: Silver Nanoparticles Functionalized With Antimicrobial Polypeptides: Benefits and Possible Pitfalls of a Novel Anti-infective Tool
Source: Front Microbiol. 2021 Dec 17;12:750556. doi: 10.3389/fmicb.2021.750556 (PMC8719061; doi:10.3389/fmicb.2021.750556)
Supplement: Supplementary file 1 [file Data_Sheet_1.PDF]

Supplementary Material

Supplementary Table 1. Example of lysoplate assay results.

**Standards and Samples**		**Lysis zones diameters (mm)**					
Concentration (μg/ml)		*500*	*250*	*125*	*62.5*	*31.2*	*15.6*
Lysozyme standards	Parallel 1	47	41	37	32	29	27
	Parallel 2	43	41	37	34	30	28
	Average	*45*	*41*	*37*	*33*	*29.5*	*27.5*
Test sample		Parallel 1	Parallel 2	Parallel 3	Parallel 4	Average	Standard deviation
AgNP-Lyz (400 μg/ml)		35	33	36	34		
Activity estimation (μg/ml)		*86.1*	*60.3*	*102.8*	*72.1*	***80.3***	***18.3***

Standard curve approximation:

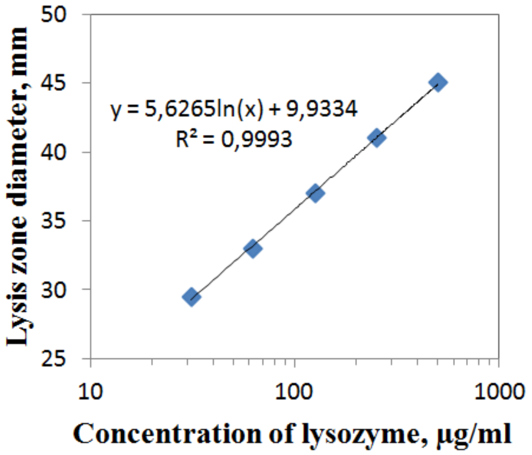

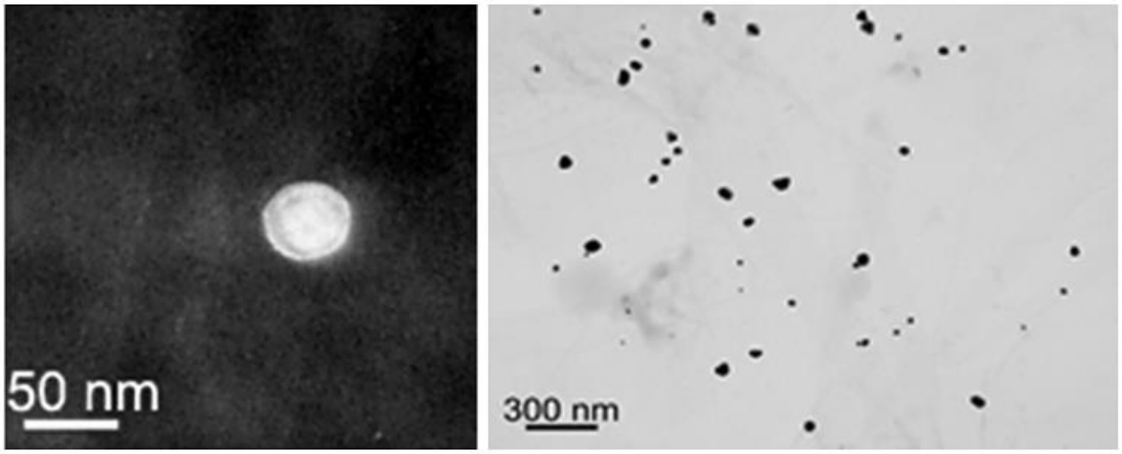

Supplementary Figure 1. Dark and bright field transmission electron microscopy (Philips EM-420) of the silver nanoparticles (AgNPs) coated with gelatin and lysozyme (AgNP-Lyz sample). Average diameter ~50 nm, average coating thickness ~14 nm.

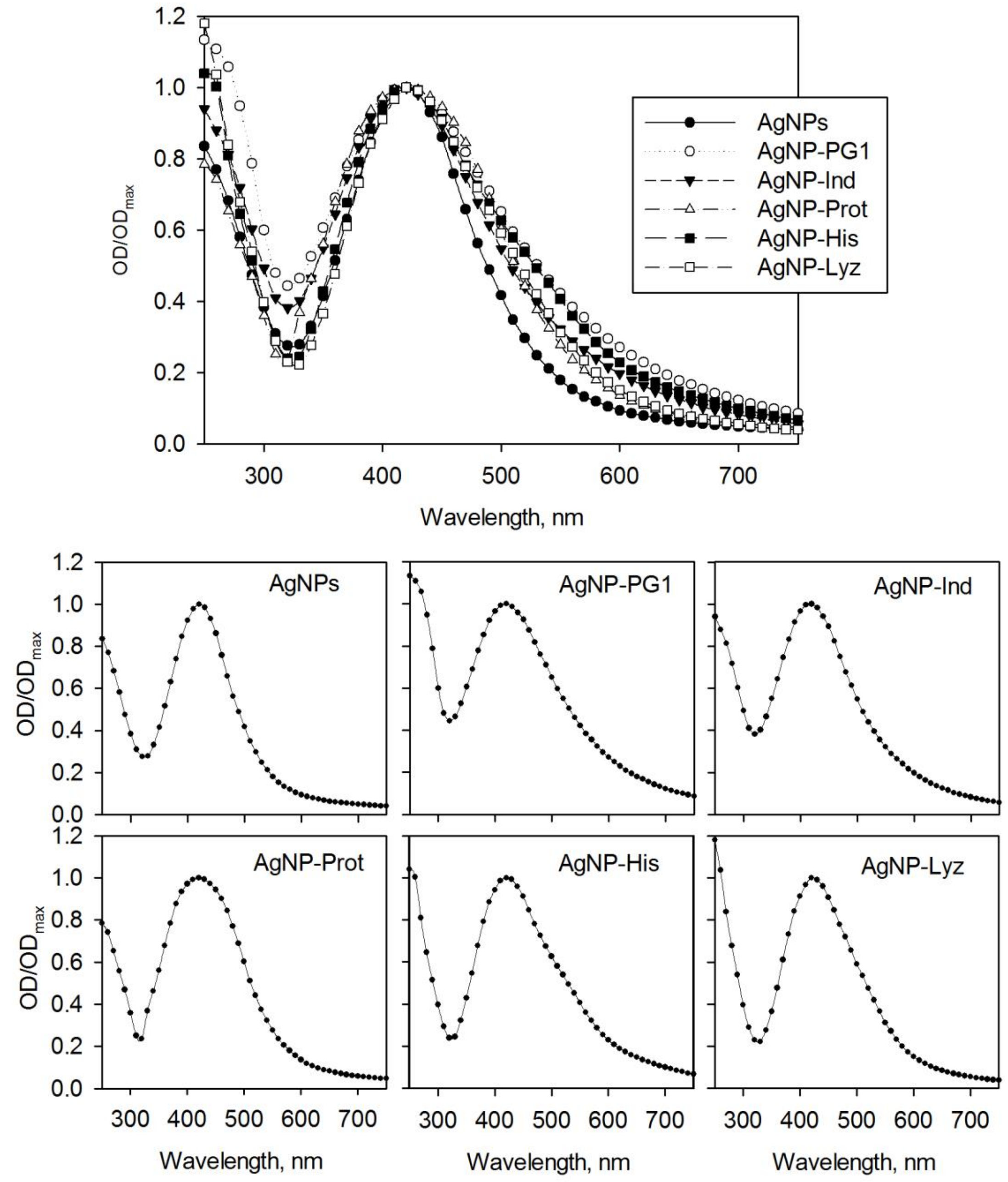

Supplementary Figure 2. Normalized UV-visible spectra of gelatin-only coated silver nanoparticles and their conjugates with antimicrobial peptides and proteins. PG1—protegrin-1, Ind—indolicidin, Prot—protamine, His—histones, Lyz—lysozyme.
